# Development of Laser Welding and Surface Treatment of Metals

**DOI:** 10.3390/ma15051765

**Published:** 2022-02-26

**Authors:** Aleksander Lisiecki

**Affiliations:** Department of Welding Engineering, Faculty of Mechanical Engineering, Silesian University of Technology, Konarskiego 18A Street, 44-100 Gliwice, Poland; aleksander.lisiecki@polsl.pl

**Keywords:** welding, laser processing, joining, tribology, cladding, properties of surface layers

## Abstract

This Special Issue on Development of Laser Welding and Surface Treatment of Metals contains as many as twenty-two research articles mainly related to the application of lasers, but also on other welding processes that may be competitive to laser technologies under specific conditions. Despite the introduction of lasers for material processing in the 1960s, the continuous development of laser devices also leads to the development and expansion of laser technology applications. This Special Issue is a compendium of knowledge in the field of fusion welding, the manufacturing of surface layers and coatings with increased wear resistance and tribological characteristics, as well as corrosion resistance and the characterization of coatings and surface layers. The topics of the presented research articles include aspects related to laser welding (eight articles), especially technological conditions, the properties of different types of joints, and analytical and numerical aspects of modelling the laser heat sources. The second dominant issue concerns laser cladding and laser surface treatment of different ferrous and nonferrous metallic and composite materials (six articles). In addition, there are interesting results of the study of fusion welding under forced cooling of the deposit or underwater conditions (four articles), results on the characterization of wear resistance coating produced by different technologies that can be competitive for laser cladding (three articles), and an original study on local strengthening of the thin-walled structure by laser treatment (one article). This Special Issue provides very wide and valuable knowledge based on theoretical and empirical study in the field of laser and fusion welding, laser and related coating technologies, characterization of coatings, and wear phenomena.

## 1. Introduction

The manufacturing and processing of materials is the basis of the modern economy and of civilization progress. Striving for sustainable development as well as limiting the harmful impact on the environment, including the reduction in CO_2_ emissions and the reduction in energy consumption, leads to a continuous increase in the effectiveness and efficiency of technological processes, machines, devices, vehicles, tools, and building structures [1,2,3].

Therefore, new materials are being developed with higher mechanical properties, higher wear resistance and thus higher operating parameters. The conventional welding methods and technologies usually do not provide satisfactory results in applications such as joining and cladding of modern materials, e.g., high strength steel, nonferrous metals and alloys, composite materials, and nanostructured and hybrid materials [1,4,5,6,7,8].

The main disadvantages of typical methods of welding and cladding such as conventional arc or plasma arc methods are excessive heat input and the relatively large volume of the molten pool. This leads to overheating of the material, internal stresses and deformations, and unfavorable grain growth or dissolution of the reinforcing phase particles, e.g., carbides or nitrides. Therefore, the attention of researchers and industry is focused on the search for methods of joining and processing materials that ensure a minimum thermal effect on the material and the ability to precisely control the amount of heat and the thermal cycle of the process [9,10,11,12,13].

One of the dynamically developed areas of material processing technology in recent years is laser material processing technology. In the area of joining materials, laser welding shows several advantages because it provides high-power densities and low diameter of the laser beam spot, and thus high penetration depth, high welding speed, and low, precise, and controllable heat input. The above features are particularly important for the joining of modern and advanced alloys such as ultra-high-strength steels (UHSS), advanced high-strength steels (AHSS), modern stainless duplex and super duplex steel, nonferrous metals, and light metals, e.g., titanium and aluminum alloys [6,11,13,14,15,16,17].

Another area of laser beam application that is currently being intensely developed is the shaping of the properties of surface layers and the production of coatings for enhanced wear characteristics such as corrosion resistance, tribological properties, abrasive resistance, thermal and mechanical fatigue resistance, and resistance to impact load [1,2,3,4,5,6,7,8,18].

In the area of surface treatment and cladding, the laser beam as a heat source is also advantageous. The most significant advantages include low and controllable heat input, and thus limited thermal impact on the substrate, reduced internal stresses, minimized distortions, high solidification and cooling rates, low penetration depth, and low dilution of the clad layer by the substrate material. Low heat inputs and related high cooling rates during laser processing are beneficial in many applications of surface treatment and cladding as they provide high quality clads and surface layers. Usually, surface layers are characterized by superior metallurgical bonding and a fine-grained and refined microstructure, which are decisive for providing superior tribological characteristics, high abrasive or erosion wear resistance, and corrosion or fatigue resistance [1,8,18].

It is worth noting that laser-made clads and surface layers show higher functional properties compared to surface layers made by plasma arc (PTA) or conventional arc cladding processes, even if the same additional materials are applied. This is due to intense grain refinement caused by very fast cooling and solidification of the molten pool, typically an order of magnitude higher than plasma (PTA) and conventional arc cladding (e.g., GTA–gas tungsten arc) [1,12].

Thanks to the advantages mentioned above, lasers are used in different processes of surface treatment such as laser surface hardening (LSH), melting (LSM), shocking (LSS), texturing (LST), alloying (LSA), cladding (LSC), remelting (LSR), and surface deposition (LSD), and in additive manufacturing processes such laser metal deposition (LMD) or selective laser sintering and melting (SLS/SLM) [1].

Continuous development of laser generators and related improvement of laser radiation and laser beam characteristics enable further development in the field of laser material processing, including laser welding and laser surface treatment. In addition to the gaseous CO_2_ and solid-state rod type Nd:YAG laser generators used for the last few decades, new generations of solid-state lasers are currently available such as high-power diode lasers (HPDL), disk lasers, and fiber lasers [1,14,15,16,17]. Modern generations of laser devices offer new or expanded technological possibilities. For example, as shown in the literature, high-power diode lasers with a square or rectangular beam spot and uniform energy distribution (multimode beam) are favorable for surface treatment applications. The design of diode lasers has been developed to the extent that today there are direct diode lasers (DDL) which emit single-mode radiation with power up to 8 kW, suitable for cutting applications. Moreover, the single-mode DDL lasers are even competitive with CO_2_ gas lasers and disk lasers, as well as fiber lasers [1,14,17,18]. 

There has also been significant progress in the design and construction of fiber generators in the last decade. Modern fiber lasers are also beneficial due to their compact design, high wall plug efficiency, low beam divergence, and possibility to focus the beam into a very small spot, even at relatively high power [18]. Moreover, these fiber lasers can provide a single-mode beam up to 20 kW, while the multimode industrial applications reach a power level of 100 kW. As a result, lasers are currently used for one-sided or two-sided welding, even for thick steel plates with a thickness up to approx. 30 mm.

Another interesting example of dynamic development in the field of material processing technology is hybrid processes. According to the definition, the hybrid process combines at least two different machining processes which must be carried out simultaneously and in the same processing area (e.g., one weld pool created during fusion welding). In the case of welding, the laser beam is often combined with the arc, e.g., laser + GMA (gas metal arc), laser + GTA (gas tungsten arc) or laser + PTA (plasma tungsten arc). Such combinations of two different heat sources allow taking the advantages of both welding methods. The laser beam is responsible for deep penetration of the material, while the arc processes provide additional heat and material to fill the gap. Consequently, it is possible to weld at high speed and ensure the correct shape of the weld at high depth. Hybrid processes are also being developed in the field of surface treatment and coating applications, ensuring higher functional parameters or the possibility of producing coatings with special properties [1,18].

## 2. Short Characterization of the Special Issue

The Materials and Methods should be described with sufficient details to allow others to replicate and build on the published results. Please note that the publication of your manuscript implicates that you must make all materials, data, computer code, and protocols associated with the publication available to readers. Please disclose at the submission stage any restrictions on the availability of materials or information. New methods and protocols should be described in detail, while well-established methods can be briefly described and appropriately cited.

This Special Issue, entitled “Development of Laser Welding and Surface Treatment of Metals”, is a complementary and valuable resource of knowledge in the following fields:

- Study of technological conditions of laser welding, investigation of test joints properties [19,20,21,22,23,24,25,26];
- Analytical and numerical models of heat sources of laser beam and arc welding processes [19,20,22,23];
- Study of laser cladding and investigations of the coating’s properties and tribological characteristics [27,28,29,30,31,32,33];
- Study and development of fusion welding processes, investigation of properties of the deposit produced under forced cooling or underwater conditions [34,35,36];
- Study of cladding, surfacing, and thermal spraying processes of wear-resistant coatings, characterization of the coatings [37,38,39];
- Study of application of local strengthening of the thin-walled structure by laser treatment [40].

In the work presented by Wang et al. [19], plates of X100 pipeline steel with a thickness of 12.8 mm were welded by a high-power laser on a robotized cell. The authors provided a quantitative correlation between thermal cycling and the microstructure of the welded joint by means of empirical and numerical study. They also showed that the effects of austenitization are more significant than those of the cooling rate on the final microstructures of the laser-welded joint of the investigated steel grade. The results of the presented work can provide scientific guidance and a reference for the simulation of the temperature field during laser welding, especially in the case of laser welding of X100 pipeline steel grade. 

Kik [20] presented different computational techniques for comparison in the case of single and multipass arc and laser beam welding processes. The numerical analyses were conducted by the SYSWELD software package and the author showed differences between the applied computational techniques, as well as the benefits and disadvantages of the applied computational techniques. The presented results of the numerical simulations may be used for preparing an efficient and fast optimization of the laser and related fusion welding processes, according to the criteria of minimizing deformations or identification of potential defects of structures. 

In the second article, Kik [23] presented new possibilities for modifying heat source models in numerical simulations of laser welding processes conducted using VisualWeld (SYSWELD) software. He proposed a modification of heat source models and methods of defining the heat introduced into welded materials using solid-state and high-power diode lasers. He provided calibration and validation of the proposed models of heat sources based on metallographic tests and thermal cycles; therefore, the presented results may be helpful for further numerical analysis of laser processing by different beam shape and energy distribution. 

The study presented by Górka [21] was focused on investigation of the influence of the basic parameters of autogenous laser welding of 700MC 10.0 mm thick plates on their microstructure and mechanical properties. He provided a comprehensive analysis of the microstructure and a detailed analysis of the phase constituents. The quality of the test joint was estimated as meeting level B, in accordance with 13919-1 standard. He concluded that in cases of S700MC steel, the analysis of the phase transformation of austenite exposed to welding thermal cycles and the value of carbon equivalent cannot be the only factors taken into consideration when assessing weldability.

The laser welding process was also investigated by Danielewski and Skrzypczyk [22]. They presented results of numerical and experimental analyses of the gaseous CO_2_ laser welding of lap joints of low-carbon structural steel. They determined hardness profiles and weld geometry for the welds produced at different parameters. They also provided detailed analysis of the metallographic structure of fusion zone (FZ) and heat-affected zone (HAZ), as well as a quality assessment of the tested joints. 

The study presented by Landowski et al. [24] concerned fiber laser welding of dissimilar butt joints between 316 L austenitic and 2304 lean duplex stainless steel plates of 8.0 mm thickness. Based on nondestructive tests and metallographic examinations, they showed that the tested joints meet the acceptance criteria for level B, in accordance with EN ISO 13919–1 standard. Finally, they proved that the elaborated procedure can be applied for welding of 316 L–2304 stainless steel plates with a thickness of 8.0 mm without ceramic backing. The results also provide valuable guidance for further research in this field.

The results of the study presented by Pańcikiewicz et al. [25] also concerned laser welding of dissimilar joints of AISI 430F (X12CrMoS17) martensitic stainless steel and AISI 304 (X5CrNi18-10) austenitic stainless steel. They provided detailed analysis of the microstructure of the base metals and the dissimilar test joint. However, the test joint meets the quality level C, in accordance with the ISO 13919-1 standard, due to gas pores in the weld metal. 

Another study on laser welding was presented by Tofil et al. [26]. They conducted a test of laser welding of thin-walled AISI 316 L steel pipes, with diameters of 1.5 and 2.0 mm, used in medical equipment. They determined the range of optimal parameters of laser welding with a SISMA LM-D210 Nd:YAG laser. The results of the study showed good joint properties with a strength of more than 75% in the thinner pipe, uniform distribution of alloying elements, and a complex dendritic structure characteristic of pulse welding. Therefore, the presented results may also be guidance for further research in this field.

The topic of laser surface treatment and cladding begins with Lisiecki’s article [27] on the study of the optical properties of surface layers produced on the titanium alloy substrate by surface melting and nitriding. The aim of this study was to determine the optical properties of the surface of titanium alloy Ti6Al4V. The specular, diffuse, and total reflection was determined for different surface conditions (roughness and oxides layer) and after laser surface treatment. The experiments were conducted for 808 nm wavelength, typical for diode lasers. The main conclusion of the study was that the distinct differences in absorption affected the heat transfer and thermal conditions of laser heating, and thereby the penetration depth during laser melting and nitriding of the titanium alloy.

A highly interesting study on laser surface modification of an aluminum alloy by boron carbides was carried out by Sroka et al. [28]. The fiber laser YLS-4000 with a maximum output power of 4.0 kW was used in this study. The effect of basic laser-alloying parameters of the foundry AlMg9 alloy with the B_4_C particle on the microstructure and chosen properties was investigated and determined. 

Another interesting study on laser cladding was performed by Li et al. [29]. In their study, the high aluminum and chromium Fe-B-C coating was produced by laser cladding on a 2Cr13 steel substrate. They investigated the microstructure and tribological characteristics. The results showed that the coating exhibited excellent wear resistance compared to the substrate. They concluded that the results could provide technical support in improving the properties of the Fe-based laser-cladded coating.

The next study, presented by Majkowska-Marzec et al. [30], is related to investigation of the mechanical and corrosion properties of a laser surface-treated titanium alloy with carbon nanotube coating. The coating was initially formed by electrophoretic deposition (EPD) and then subjected to laser treatment. The authors pointed to some advantages of such a coating. The main advantages were increased corrosion resistance, a lack of surface cracks, increased strength, and favorable contact angles between 46° and 82° (resulting in hydrophilic surfaces suitable for cell adhesion).

A comparative study of laser and plasma cladding was presented by Czupryński et al. [31]. The coatings of Inconel 625 on the 16Mo3 steel pipes were produced by plasma (PTA) cladding and high-power direct diode laser (HPDDL) cladding. The authors determined the quality of the coating by NDT testing, metallographic examinations, and microstructure analysis. They concluded that both tested and compared methods can provide high-quality protective coatings that may be operated at elevated temperatures up to 625 °C. 

Another study on the effect of laser treatment on tribological properties was presented by Lubas et al. [32]. They analyzed the influence of different engine oils on the tribological parameters of sliding couples with a laser-borided surface layer produced on specimens of AISI 5045 steel, by laser remelting of a surface layer coated with amorphous boron. The tribological and wear characteristics were investigated on the pairs of AISI 5045 steel and SAE-48 bearing alloy, lubricated with 5W-40 and 15W-40-type engine oils. The detailed analysis of wear mechanisms was identified and described. 

The thematic series on laser surfacing and cladding ends with the article presented by Lisiecki and Ślizak [33] on hybrid laser deposition of composite coatings at cryogenic conditions. This study demonstrated the effect of forced and localized cooling by a nitrogen vapor stream under cryogenic conditions during laser deposition of WC-Ni powder on the geometry and microstructure of clad layers and the dry sliding wear resistance of the coatings. Additionally, the quantitative relationship between heat input, cooling conditions, and carbide grain size distribution, as well as carbide share in relation to the matrix, was determined. It was shown that the novel demonstrated technique of localized forced cooling during laser cladding has advantageous effects. The forced localized cooling provides approximately 20% lower penetration depth and dilution, decreases tendency for tungsten carbide decomposition, and provides more uniform distribution and a higher share of massive eutectic W_2_C-WC carbides across the coating. 

Another very novel and interesting subject related to arc welding processes was presented by Szczucka-Lasota and Szymczak et al. [34,35]. The study presented in two articles was related to the application of a novel and patented technique of localized cooling of the weld by micro-jet streams. The first presented article is focused on the influence of processing conditions, especially localized cooling on the properties of the weld of Docol 1200 M steel. They found that the novel technique of cooling can provide a high fatigue limit at the level of at least 480 MPa. The second article presented by Szymczak et al. [35] is related to a similar issue but a different type of steel: S960MC sheet of 2.0 mm thickness. In this case, the Wöhler S–N curve of the weld was determined, indicating that the value of the fatigue limit of the tested weld was 100 MPa. The weld at the Union NiMoCr welding wire was indicated as the joint with the highest resistance on static and fatigue loadings.

Some manuscripts dealt with other arc processes and technologies, but the presented solutions were very innovative. An example of a highly interesting subject was presented by Tomków et al. [36]. They investigated underwater welding in terms of the role of the bead sequence during welding under such conditions. The test beads were produced by covered electrodes with the use of normalized S355G10 + N steel. They reported that welding in the underwater environment carries many problems related to arc stability, and thus the properties of the welds. Moreover, they revealed the effects of refining and tempering the microstructure in heat-affected zones of the previous beads, which resulted in a reduction in hardness. 

The study presented by Mician et al. [37] was related to the influence of bead sequence on the deposit and heat-affected zone properties during arc surfacing. The process applied to surfacing was gas metal arc welding (GMAW) and the substrate plate was an S355 steel plate. The authors provided a comparative analysis of structure and hardness, considering the thermal impact of the bead sequence. As a result of the study, the most favorable sequence in terms of structure and hardness distribution, maximum hardness, and range of hardness has been established. 

The second study presented by Czupryński [38] was focused on flame spraying of aluminum coatings reinforced by carbon nanotubes as an alternative for laser cladding technology. The microstructure of the coatings was analyzed, as well as abrasion and erosion resistance. He concluded that the flame spraying of aluminum coatings reinforced with the carbon nanotubes can be an effective alternative for laser cladding technology. Moreover, such coatings can be implemented in the automotive industry for the production of components characterized by high strength, wear resistance, good thermal conductivity, and low density.

The next article provided by Czupryński [39] presented results of the investigations of abrasive wear characteristics of wear-resistant plates made by cladding with innovative tubular electrodes with a metallic core and an experimental chemical composition. Detailed analyses of microstructure and wear tests of different reference plates were conducted. The author showed a high metal–mineral abrasive wear resistance of the deposit produced by the experimental tubular electrode. 

Another original issue was presented in the study by Kapustynskyj et al. [40]. They focused on the numerical and analytical study of the effects of laser surface processing of thin-plate low-carbon structural steel. The authors developed the original analytical methodology of the estimation of the cross-section area of the laser-processed metal, that can be applied to the choosing of a reasonable distance between the centers of the laser-processed tracks. The results of the experimental and finite element numerical and analytical analyses showed that the laser treatments of the surface of the steel plate increase the yield point of the laser-processed region, as well as the axial and flexural stiffness of the plate. 

## 3. Concluding Remarks

This Special Issue was very successful due to the valuable articles that were submitted, the wide variety of topics and research problems that were undertaken, and in-depth analysis of the state of the art in different fields of laser and related processes. This Special Issue contains a significant number of research articles (22 in total); however, the entire number of manuscripts submitted to this Special Issue was half that higher. Unfortunately, some of the manuscripts have not gone through a very rigorous review process. Such a large interest and quantity of articles shows the importance of the issue and themes. It should also be emphasized that each research article in the field of materials processing is the result of tedious, long-term, and interdisciplinary research, usually conducted by a team of scientists. 

The topics of the articles were mainly focused on laser welding, laser cladding, and laser surface treatment, as well as related processes of fusion welding and manufacturing of coatings. 

The wide range of experimental, numerical, or analytical study conducted and presented by 58 authors, representing 19 academic or research centers and 3 industrial centers from 5 countries (Poland, China, Slovakia, Lithuania, and Ukraine) and 16 cities, proved the topicality and importance of this Special Issue. 
